# Long noncoding RNA Ftx regulates the protein expression profile in HCT116 human colon cancer cells

**DOI:** 10.1186/s12953-022-00187-1

**Published:** 2022-04-30

**Authors:** Ruzhen Jia, Lulu Song, Zhiqiang Fei, Chengyong Qin, Qi Zhao

**Affiliations:** 1 Department of Gastroenterology, Shandong Provincial Hospital, Cheeloo College of Medicine, Shandong University, Lixia District, No. 44, Wenhua West Road, Jinan, 250021 Shandong China; 2 Department of Gastroenterology, Shandong Provincial Hospital Affiliated to Shandong First Medical University, Jinan, 250021 Shandong China; 3 Department of Gastroenterology, Shandong Second Provincial General Hospital, Jinan, 250022 Shandong China

**Keywords:** Long noncoding RNA, Colorectal cancer, Proteome, Metastasis

## Abstract

**Background:**

The long noncoding RNA (lncRNA) five prime to Xist (Ftx) is involved in distant metastasis in colorectal cancer (CRC). This study aimed to investigate Ftx alteration-induced proteomic changes in the highly metastatic CRC cell line HCT116.

**Methods:**

Tandem mass tag (TMT)-based proteomics analysis was performed to detect the differential protein expression in Ftx-overexpressing and Ftx-silenced HCT116 cells. The differentially expressed proteins were classified and characterized by bioinformatics analyses, including gene ontology (GO) annotation, GO/Kyoto Encyclopedia of Genes and Genomes (KEGG) pathway/protein domain enrichment analyses, as well as hierarchical clustering. A total of 5471 proteins were quantified, and the proteins with |fold change|≥ 1.2 and *p* < 0.05 were identified as differentially expressed proteins in response to Ftx overexpression or silencing.

**Results:**

The bioinformatics analyses revealed that the differentially expressed proteins were involved in a wide range of GO terms and KEGG signaling pathways and contained multiple protein domains. These terms, pathways, and protein domains were associated with tumorigenesis and metastasis in CRC.

**Conclusions:**

Our results indicate that the alteration of Ftx expression induces proteomic changes in highly metastatic HCT116 cells, suggesting that Ftx and its downstream molecules and signaling pathways could be potential diagnostic biomarkers and therapeutic targets for metastatic CRC.

## Background

Colorectal cancer (CRC) is the third most common cancer and the fourth leading cause of cancer deaths worldwide, with a median survival of 20 months and over 600,000 deaths per year [1]. Distant metastasis is the major cause of CRC-related deaths. About 20% of patients with CRC present distant metastases at the initial diagnosis, and approximately 50% of patients develop metastases within five years [2, 3]. Therefore, there is a critical need to elucidate the underlying mechanism of CRC metastasis and to identify novel therapeutic targets for antimetastasis treatment. CRC metastasis occurs through a sequential, multistep process that involves multiple factors and mechanisms [4]. Recent studies have used proteomics analysis to identify differentially expressed proteins in metastatic CRC compared with primary CRC [5, 6], providing some useful information for CRC diagnosis and treatment. However, the upstream regulator that causes proteomic alterations in metastatic CRC remains unexplored.

Long noncoding RNAs (lncRNAs) represent a class of noncoding transcripts containing more than 200 nucleotides [7]; they act as architectural RNAs, microRNA sponges, or regulators of multiple cellular functions, such as epigenetic modification, gene transcription, and posttranscriptional processes [8]. Dysregulation of lncRNAs is associated with various human diseases, including cancer [9] and plays a critical role in tumorigenesis, tumor progression, and metastasis [10]. The lncRNA five prime to Xist (Ftx) is a well-conserved 2300-bp RNA encoded within the X-inactivation center on the X chromosome [11]. Previous studies have shown that Ftx facilitates tumor growth in many types of cancer, including hepatocellular carcinoma, gastric cancer, and CRC [12–14]. Guo et al*.* have demonstrated that Ftx expression is significantly upregulated in CRC tissue compared with normal tissue and that it positively correlates with the tumor grade, pathologic stage, and overall survival of patients with CRC. Ftx overexpression promotes cell proliferation, migration, invasion, and colony formation in multiple CRC cell lines [15]. Furthermore, Yang et al*.* have found that Ftx expression is positively associated with distant metastasis in CRC. In addition, knockdown of Ftx markedly suppresses the malignant behavior of CRC cells in vitro and inhibits CRC growth and distant metastasis in vivo [14]. Although miRNA-215 and vimentin have been shown to mediate the oncogenic role of Ftx in CRC, the downstream molecules and signaling pathways of Ftx in CRC tumorigenesis and metastasis remain unknown. A recent study has demonstrated that overexpression or knockdown of Ftx alters the proteomic profile of gastric cancer cells, inducing the on/off switching of gastric carcinogenesis [16]. Therefore, we hypothesized that the alteration in Ftx expression might induce metastasis-related proteomic changes in CRC cells.

To test our hypothesis, we performed a tandem mass tag (TMT)-based proteomics assay in highly metastatic HCT116 CRC cells [17, 18] in response to Ftx overexpression or silencing. Using bioinformatics analysis, we identified and characterized a panel of differentially expressed proteins and signaling pathways that are potential downstream targets of Ftx in CRC. These findings may provide valuable clues toward a better understanding of the molecular mechanisms underlying CRC metastasis.

## Methods

### Cell culture and transfection

HCT116 cells were obtained from the Cell Bank of the Chinese Academy of Science (Shanghai, China) and cultured in Dulbecco’s modified Eagle medium (HyClone, Marlborough, MA, USA) supplemented with 10% fetal bovine serum in a humidified atmosphere of 5% CO_2_ at 37 °C. Lentiviral vectors (Shanghai GeneChem, Shanghai, China) expressing Ftx, mock control, small interfering RNA (shRNA) of Ftx, or scrambled shRNA were constructed, according to the manufacturer’s protocol. The shRNA was amplified with the following primer for sequencing: 5′-CcggTTGGCATAAAGTGTAGTGTAACTCGAGTTACACTACACTTTATGCCAATTTTTg-3′, where the loop is bold underlined. The shRNA sequencing data were deposited at the NCBI (https://www.ncbi.nlm.nih.gov/nuccore/NR_028379.1). HCT116 cells were transfected with sh-Ftx and screened with puromycin (2 μg/mL) to produce cell lines with stably interfered expression of the lncRNA Ftx, whereas HCT116 cells stably transfected with sh-NC were used as its mock control. To obtain cell lines stably overexpressing the lncRNA Ftx, HCT116 cells were transfected with Ftx and screened with puromycin (2 μg/mL), whereas HCT116 cells transfected with Ftx-NC were used as its mock control. The stable lncRNA Ftx-interfered or -overexpressing cell lines were validated by real-time PCR with the following primers (forward: 5′-GAATGTCCTTGTGAGGCAGTTG-3′ and reverse: 5′-TGGTCACTCACATGGATGATCTG-3′).

### TMT labelling

**Fig. 1:**
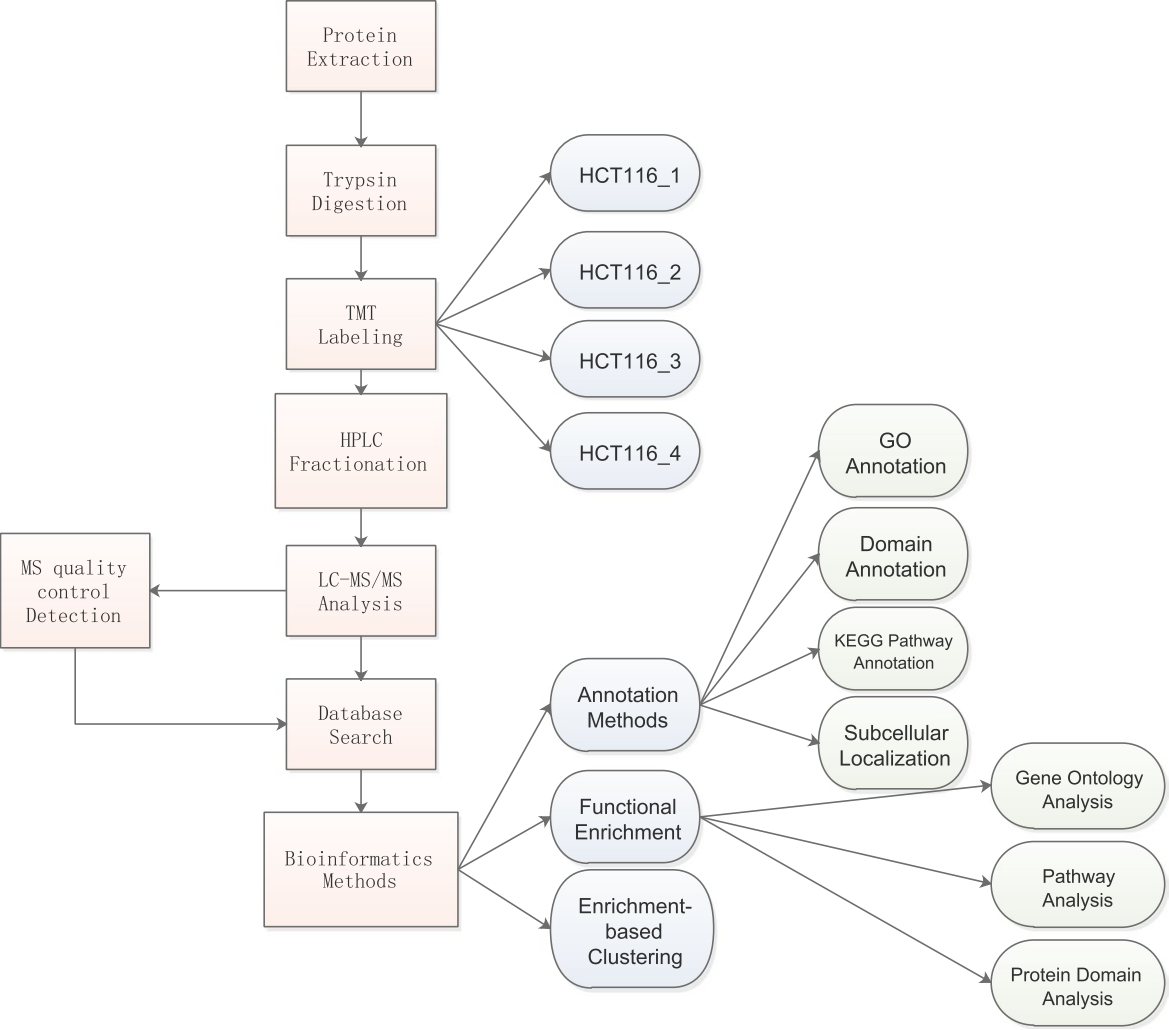
Schematic workflow of the TMT labeling procedures

Ftx-overexpressing or Ftx-silenced HCT116 cells were lysed using lysis buffer containing 8 M urea, 1% protease inhibitor, and 2 mM EDTA. The lysates were obtained by centrifugation at 12,000 g and 4 °C for 10 min. Proteins in the final lysate were quantified using a bicinchoninic acid kit (Beyotime Biotechnology, Shanghai, China). The proteins were then digested with trypsin (Promega, Madison, WI, USA) at a trypsin-to-protein ratio of 1:50 overnight at 37 °C, followed by an additional 4-h digestion at a trypsin-to-protein ratio of 1:100. The tryptic peptides were desalted with a Strata X C18 cartridge (Phenomenex, Torrance, CA, USA) and freeze-dried *in vacuo*. Each sample was resuspended in 0.5 M triethylammonium bicarbonate (TEMB) solution and labeled using a TMT 6-plex Isobaric Mass Tagging Kit (Thermo Fisher Scientific, Waltham, MA, USA). As shown in Fig. 1, briefly, one unit of the TMT labeling reagent was thawed and dissolved in anhydrous acetonitrile for 5 min with occasional vortexing. The samples HCT116_1, HCT116_2, HCT116_3 and HCT116_4 were labeled as 126, 127,130 and 131, respectively. To each tube containing resuspended peptides was added 41 µL of resuspended TMT reagent, and the mixture was gently mixed and incubated at room temperature for 2 h. Then the samples were mixed in a ratio of 1:1, desalted using a C18 column, and freeze-dried *in vacuo* for mass spectrometry.

### Liquid chromatography–mass spectrometry

The TMT-labelled peptides were fractionated using an Agilent 300Extend C18 system (Agilent Technologies, Santa Clara, CA, USA). The samples were then resuspended in 0.1% (v/v) formic acid/water solution (mobile phase A) and separated using an EASY-nLC 1000 UHPLC system (Thermo Fisher Scientific). Mobile phase B was an aqueous solution containing 0.1% formic acid and 90% acetonitrile. After separation, the samples were analyzed by a Q Exactive plus mass spectrometer (Thermo Fisher Scientific). The precursor and fragment ions were detected and analyzed by an Orbitrap mass analyzer.

### Protein identification and database search

The secondary-order mass spectra data was searched in the protein sequence database SwissProt Human (20,130 sequences) using Maxquant (v1.5.2.8). An anti-database was added to calculate the false positive rate (FDR) caused by random matching, and a common contamination library was added to the database to eliminate the contaminating protein from the impact identification results. Search parameters were as follows: the digestion method is Trypsin / P; the number of missed cut sites is 2; the minimum length of the peptide is 7 amino acid residues; the maximum number of peptide modifications is set to 5; the first-level precursor ion mass tolerance of First search and Main search was 20 ppm and 5 ppm, respectively, and the secondary fragment mass tolerance was 0.02 Da. The cysteine alkylation is set as a fixed modification, and the variable modification is the oxidation of methionine and the acetylation of the N-terminus of the protein. The quantitative method was set to TMT-6plex with the false discovery rate (FDR) of 1% for identification of protein and peptide-to-spectrum matches (PSM).

### Identification of differentially expressed proteins

The peptide mass spectra were processed to generate the raw data and then analyzed with the protein sequence database Maxquant (v1.5.2.8). The proteins with an adjusted *p*-value < 0.05 and |fold change|≥ 1.2 were identified as differentially expressed proteins.

### Bioinformatics analysis

The differentially expressed proteins were classified by gene ontology (GO) secondary annotation. The subcellular distribution of the proteins was predicted by Wolf PSORT (https://wolfpsort.hgc.jp/). The protein pathways were annotated using the Kyoto Encyclopedia of Genes and Genomes database (KEGG; http://www.genome.jp/kegg/). The protein domain was identified using the InterPro database (http://www.ebi.ac.uk/interpro/). Protein–protein interaction analysis of differentially expressed proteins were analyzed by the search tool for the retrieval of interacting genes (STRING, https://string-db.org). The highly correlated DEGs were mapped into STRING to identify the potential interactions and relationships among these genes, which were detected using a confidence score ≥ 0.7 and the maximum number of interactors = 0 as the cut-off criteria.

### Statistical analysis

Data were statistically analyzed by GraphPad Prism 5 software (GraphPad, San Diego, CA, USA). Differentially expressed proteins were identified by the Student’s t test, and a *p*-value < 0.05 was considered statistically significant.

## Results

### Ftx regulates the protein expression profile in HCT116 cells

**Fig. 2:**
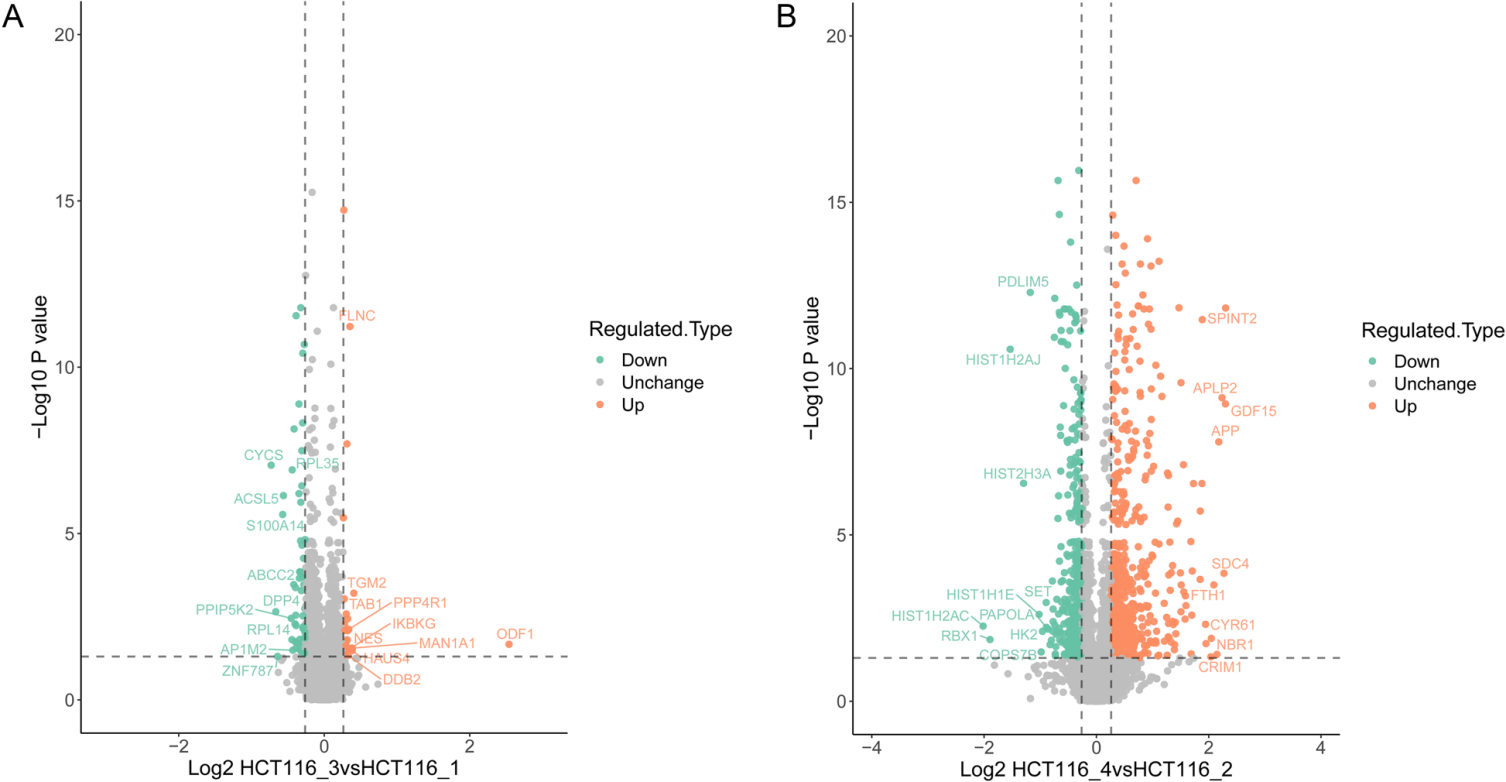
Volcano plots of differentially expressed proteins in HCT116_3 vs. HCT116_1 (**A**) and HCT116_4 vs. HCT116_2 (**B**)

To investigate whether Ftx plays a role in CRC metastasis, we performed a TMT-based proteomic assay in highly metastatic HCT116 CRC cells. Using the protein sequence database Maxquant, we identified and quantified a total of 5471 proteins. After statistical analysis, we found 556 upregulated and 367 downregulated proteins in Ftx-silenced HCT116 cells, as well as 25 upregulated and 62 downregulated proteins in Ftx-overexpressing cells (|fold change|≥ 1.2, *p-value* < 0.05) (Fig. 2A and 2B). Representative differentially expressed proteins are shown in Table 1. We noticed that the protein expression of nestin (NES), which is typically overexpressed in cancer cells [19, 20], was significantly enhanced in response to Ftx overexpression; while it was attenuated in response to Ftx silencing. In contrast, the expression of the proapoptotic protein cytoplasmic FMR1-interacting protein 2 (CYFIP2) [21] was significantly attenuated in Ftx-overexpressing cells but enhanced in Ftx-silenced cells. These findings suggest that Ftx might play an oncogenic role in CRC by regulating the expression of multiple cancer-related proteins.

**Table 1:** Representative differentially expressed proteins in Ftx-overexpressing or Ftx-silenced HCT116 cells

Protein accession	Ratio of expression in Ftx-overexpressing cells to NC	Alteration	*p-value*	Ratio of expression in Ftx-silenced cells to NC	Alteration	*p-value*	Gene name	TMT score
P48681	1.25	Up	8.34E-03	0.802	Down	1.18E-02	NES	10.302
Q86TB9	1.211	Up	9.18E-04	0.79	Down	3.72E-03	PATL1	13.762
Q96F07	0.823	Down	3.16E-02	1.541	Up	5.94E-03	CYFIP2	8.8501
P52630	0.82	Down	7.30E-03	2.379	Up	3.02E-03	STAT2	10.721
P10620	0.772	Down	1.98E-02	1.574	Up	5.79E-04	MGST1	12.703
P45877	0.758	Down	1.77E-02	1.964	Up	1.71E-02	PPIC	9.5352
Q9HCY8	0.673	Down	2.68E-06	1.329	Up	1.80E-03	S100A14	22.228

*NC* negative control

### GO secondary annotation and subcellular localization of differentially expressed proteins

**Fig. 3:**
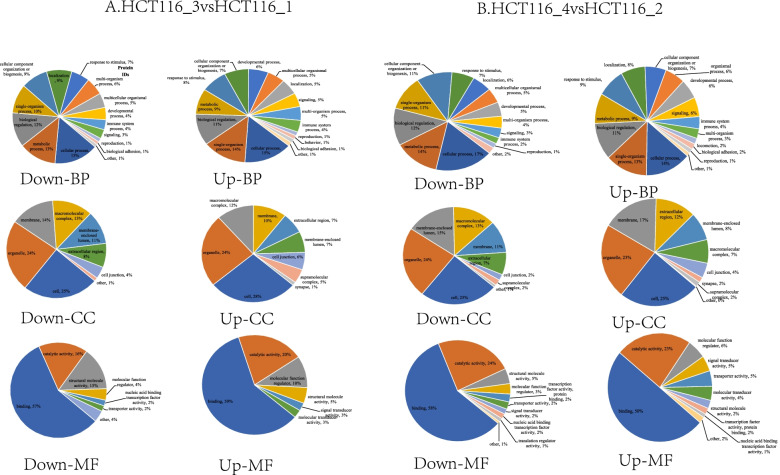
GO functional terms of differentially expressed proteins in HCT116_3 vs. HCT116_1 (**A**) and HCT116_4 vs. HCT116_2 (**B**)

**Fig. 4:**
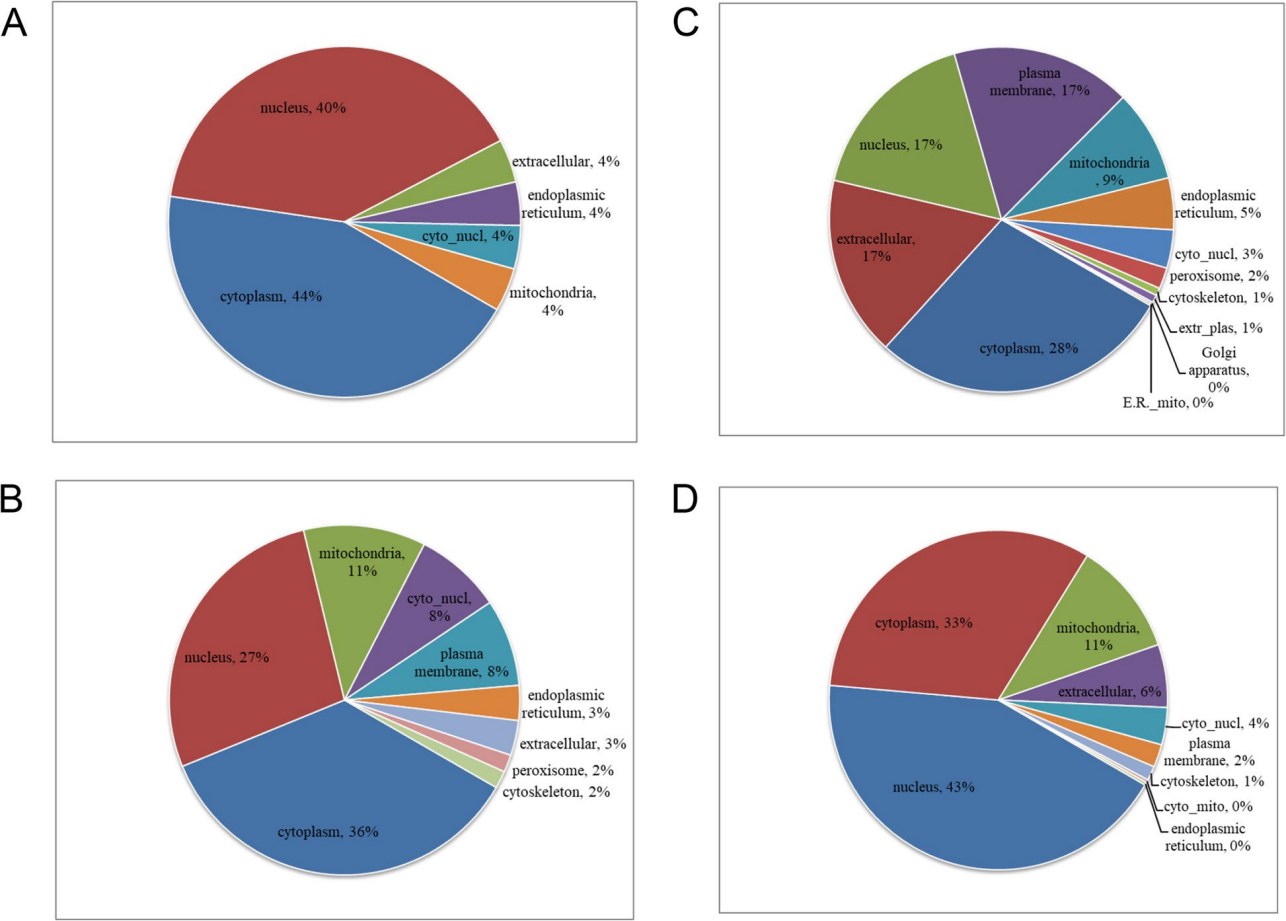
Subcellular localization of differentially expressed proteins in response to Ftx overexpression or silencing. The pie charts summarize the distribution of significantly upregulated (**A** and **C**) and downregulated (**B** and **D**) proteins in Ftx-overexpressing (**A** and **B**) or Ftx-silenced (**C** and **D**) HTC116 cells. Ftx, five prime to Xist

Next, we performed GO secondary annotation to classify the differentially expressed proteins induced by Ftx overexpression or silencing. As presented in Fig. 3, these proteins were mainly classified as cellular or single-organism processes, cell and organelle components, and molecular binding function. Wolf PSORT predicted that in Ftx-overexpressing HCT116 cells, most of the differentially expressed proteins were distributed in the cytoplasm or nucleus (Fig. 4A and 4B). In Ftx-silenced cells, the upregulated proteins were mainly located in the cytoplasm, extracellular region, nucleus, or plasma membrane; whereas the downregulated proteins were mainly located in the cytoplasm or nucleus (Fig. 4C and 4D). These results suggest that Ftx dysregulation induces the differential expression of proteins involved in a wide range of biological processes, cellular components, and molecular functions in CRC cells.

### GO, KEGG, and protein domain enrichment analyses

**Fig. 5:**
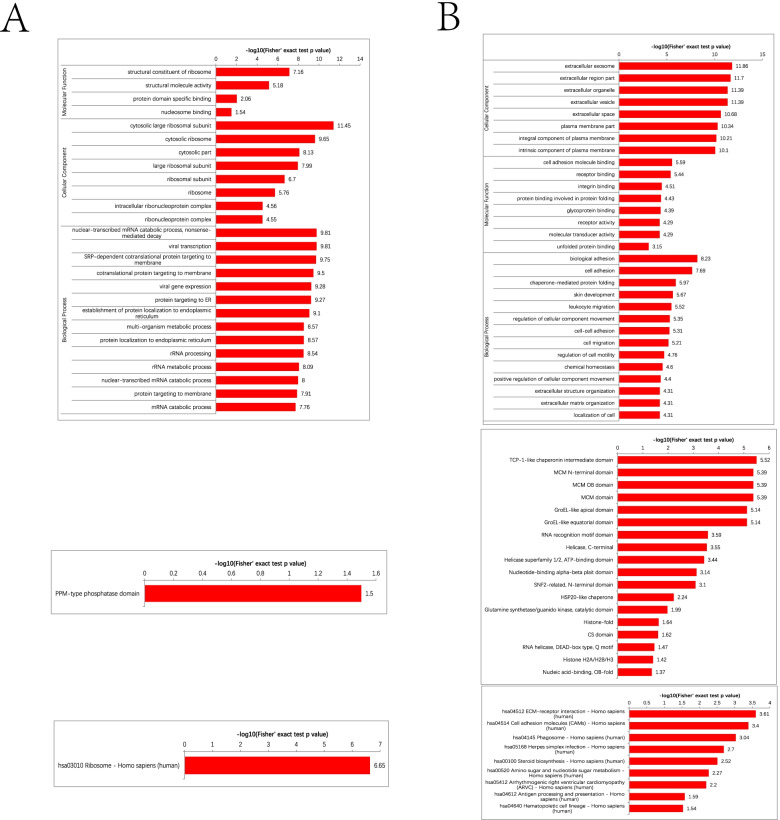
GO, KEGG, and protein domain enrichment analyses. GO, KEGG, and protein domain enrichment analyses were performed to characterize the differentially expressed proteins in Ftx-overexpressing (**A**) or Ftx-silenced (**B**) HTC116 cells. GO, gene ontology; KEGG, Kyoto Encyclopedia of Genes and Genomes

To further investigate the functional significance and to characterize the differentially expressed proteins induced by Ftx overexpression or silencing, we performed GO, KEGG pathway, and domain enrichment analyses. According to the GO enrichment analysis (Fig. 5A and 5B), the differentially expressed proteins induced by Ftx overexpression were mostly enriched in the molecular function of structural constituent ribosome, whereas those induced by Ftx silencing were mostly enriched in cell adhesion molecule binding. In terms of the cellular components, the differentially expressed proteins were mostly enriched in the cytosolic large ribosome subunit due to Ftx overexpression and the extracellular exosome due to Ftx silencing. In terms of the ontology of biological function, the differentially expressed proteins were mainly enriched in the nuclear-transcribed mRNA catabolic process due to Ftx overexpression and biological adhesion due to Ftx silencing.

Moreover, the KEGG pathway and protein domain enrichment analyses showed that Ftx overexpression induced the differential expression of proteins related to the ribosome pathway and the protein phosphatase Mg^2+^- or Mn^2+^-dependent (PPM)-type phosphatase domain, whereas Ftx knockdown altered the expression of proteins related to the extracellular matrix (ECM)-receptor interaction pathway and the immunoglobulin-like fold domain.

The differential proteins in the Ftx overexpression group and the Fxt interference group were identified to select core proteins for the protein–protein interaction network. The Ftx overexpression resulted in interacting with ribosomal (RPL) protein, supporting the KGEE results that the Ftx overexpression affected the RPL pathway. In the protein–protein interaction network, Ftx interference led to a variety of protein interactions. Among them, proteins such as ECM, PP2A, HSP90, Bcl-xl, and ERK were participated in the PI3K/Akt pathway.

### Hierarchical clustering analysis

**Fig. 6:**
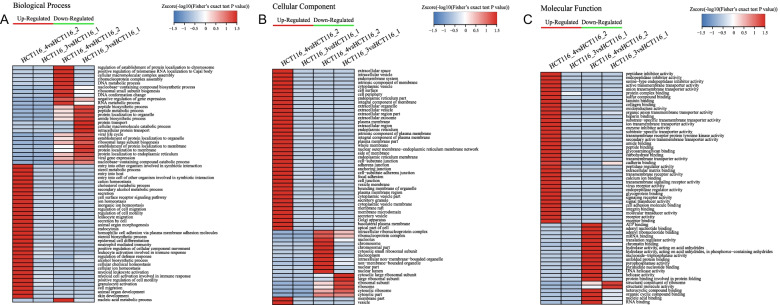
GO enrichment-based cluster analysis. The differentially expressed proteins were classified by GO annotation-based cluster analysis according to biological process (**A**), cellular compartment (**B**), and molecular function (**C**). HCT116_1, HCT116 cells transfected with empty lentiviral vectors; HCT116_2, HCT116 cells transfected with lentiviral vectors expressing scrambled shRNA; HCT116_3, HCT116 cells transfected with lentiviral vectors overexpressing Ftx; HCT116_4, HCT116 cells transfected with lentiviral vectors overexpressing shRNA against Ftx

Next, we performed clustering analyses based on the GO, KEGG, and domain enrichment data. The GO enrichment-based clustering analysis (Fig. 6A–C) showed that the downregulated proteins due to Ftx overexpression were significantly enriched in biological processes such as protein transport and protein localization, cellular components such as ribosome and ribosomal subunits, and molecular functions such as structural constituent of ribosome. The downregulated proteins due to Ftx silencing were significantly enriched in biological processes such as DNA metabolic processes and ribosomal small subunit biogenesis, cellular components such as ribonucleoprotein complex and chromosome, and molecular functions such as mRNA binding and chromatin binding. On the other hand, the upregulated proteins due to Ftx silencing were significantly enriched in biological processes such as regulation of cell migration and mobility, cellular components such as cell junction and focal adhesion, and molecular functions such as collagen binding and cadherin binding. No clusters were observed in the upregulated proteins due to Ftx overexpression.

**Fig. 7:**
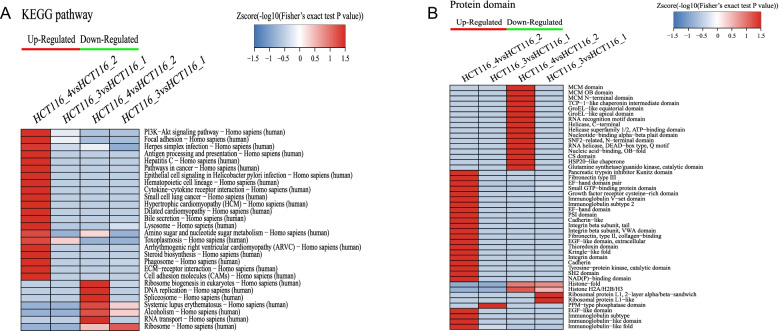
KEGG pathway- and protein domain-based cluster analyses. **A** KEGG pathway-based cluster analysis. **B** Protein domain-based cluster analysis. HCT116_1, HCT116 cells transfected with empty lentiviral vectors; HCT116_2, HCT116 cells transfected with lentiviral vectors expressing scrambled shRNA; HCT116_3, HCT116 cells transfected with lentiviral vectors overexpressing Ftx; HCT116_4, HCT116 cells transfected with lentiviral vectors overexpressing shRNA against Ftx

According to the KEGG-based clustering analysis, we found that Ftx overexpression downregulated the ribosome pathway. Ftx silencing downregulated ribosome biogenesis, while it upregulated the phosphoinositide 3-kinase–Akt and ECM-receptor interaction pathways (Fig. 7A). Meanwhile, domain-based clustering analysis demonstrated that the upregulated proteins due to Ftx silencing contained cadherin and cadherin-like domains; whereas the downregulated proteins contained the minichromosome maintenance (MCM) domain. On the other hand, after Ftx overexpression, the upregulated proteins contained the PPM-type phosphatase domain; whereas the downregulated proteins contained the ribosomal protein (RP) L1-like domain (Fig. 7B). Taken together, these data suggest that dysregulation of Ftx expression could significantly change the abundance or activity of multiple proteins and signaling pathways that are involved in the tumorigenesis and metastasis of CRC.

**Fig. 8:**
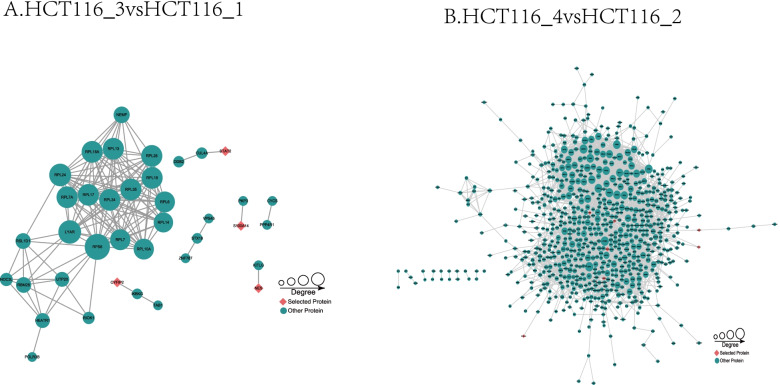
The protein–protein interaction network of differentially expressed proteins in in HCT116_3 vs. HCT116_1 (**A**) and HCT116_4 vs. HCT116_2 (**B**)

The protein–protein interaction network was constructed by the STRING tool, and the most significant molecular interaction modules (Fig. 8A and 8B) were mined by the Cytoscape tool using the following cutoff criteria: e-value = 1e-10 and string score > 700, thus showing interactions of these selected proteins with others.

## Discussion

Compared with transcriptome analysis, quantitative proteome analysis can more accurately reflect the changes in protein abundance [22] and has been widely used to identify potential biomarkers and specific protein pathways involved in cancer [23]. The lncRNA Ftx plays an important role in regulating the malignant behavior of different cancer cells [12, 14, 15, 24]; however, previous studies investigating the underlying mechanisms of Ftx have mainly focused on its interaction with microRNAs. It remains unknown whether Ftx can regulate the expression of proteins involved in cancer development. In this study, by analyzing the proteomic changes induced by Ftx alteration in HCT116 cells, we identified a panel of potential target proteins downstream of Ftx and characterized these proteins using bioinformatics approaches and identified multiple possible signaling pathways downstream of Ftx in CRC cells. Furthermore, we explored the downstream miRNAs that the lncRNA Ftx might act on with the iRDB-MicroRNA Target Prediction Database (http://mirdb.org). In total, 75 predicted miRNAs targets were identified, among which miR-608 [25], miR-21 [26], and miR-655 [27] play an important role in the occurrence and development of CRC. In addition, target matching between the lncRNA Ftx and the two important proteins NES and CYFIP2 with altered expression showed more than 88.80% homology within one gene sequence. Therefore, lncRNA might directly or indirectly target mRNA through miRNA to regulate the protein expression profile.

NES is a type VI intermediate filament protein that is widely expressed in progenitor cells [28, 29]. NES is also detectable in many cancer cell lines, playing an essential role in cancer cell proliferation, migration, and invasion [19, 20]. Li et al*.* have demonstrated that human CRC tissue has a significantly higher mRNA expression of NES compared with normal tissue. In addition, knockdown of NES inhibits the cell proliferation and migration of SW480 and HCT116 CRC cells, suggesting that NES is required for CRC growth and metastasis [30]. However, the regulation of NES in cancer remains unclear. In the present study, we found that Ftx overexpression enhanced the protein expression of NES, whereas Ftx knockdown attenuated the protein expression of NES; these findings suggest that Ftx might be an upstream regulator of NES in CRC. Epigenetic regulation and transcription factors like sex determining region Y-box transcription factor 2 and steroidogenic factor 1 are responsible for NES regulation [31, 32]. We speculate that Ftx might regulate NES expression in CRC cells through epigenetic modification and transcriptional modulation.

On the other hand, Ftx overexpression inhibited CYFIP2 expression, whereas Ftx knockdown promoted CYFIP2 expression in HCT116 cells. As a p53-inducible proapoptotic protein [21], CYFIP2 mediates caspase-3- and poly(ADP-ribose) polymerase-dependent apoptosis in SW480 colon cancer cells, suggesting that CYFIP2 is required for cell apoptosis in CRC [33]. Despite a lack of direct evidence that inhibition of CYFIP2 contributes to CRC metastasis, a previous study has shown that CYFIP1, which shares 88% amino acid sequence similarity with CYFIP2, was lost in the majority of invasive colon adenocarcinoma tissues compared with noninvasive adenoma tissues. In addition, low expression of CYFIP1 was significantly correlated with vascular invasion in patients with CRC [34]. Therefore, we speculate that Ftx overexpression attenuates the protein expression of CYFIP2, thereby facilitating CRC invasion and metastasis.

To further understand the biological significance of the proteins differentially expressed in Ftx-overexpressing or Ftx-silenced CRC cells, we conducted GO, KEGG, and protein domain enrichment analyses as well as hierarchical clustering. We noticed that the differentially expressed proteins in response to Ftx alteration were closely associated with ribosomal function, ribosome pathways, and the RP L1-like domain, suggesting that Ftx might contribute to CRC development through regulating RPs, ribosome biogenesis, and global protein synthesis. The expression pattern of RPs has been associated with CRC development and metastasis. For example, the mRNA level of RPP0 was significantly increased in primary CRC compared with the normal control and was further elevated in CRC liver metastasis compared with primary liver tumor tissue [35]. In contrast, some RPs, such as RPL5 and L6, have been shown to be downregulated in metastatic CRC compared with nonmetastatic tumors [36]. Despite lacking a protein-coding capacity, a large percentage of lncRNAs are associated with ribosomes [37]. Therefore, Ftx might modify the translational activity of ribosomes to induce a broad range of effects on downstream effectors involved in CRC metastasis.

Liu et al. have reported that Ftx inhibits cell proliferation and metastasis in hepatocellular carcinoma by binding to the DNA replication licensing factor MCM2 and microRNA-374a [38]. Our study showed that downregulated proteins due to Ftx silencing contained the MCM domain and were significantly associated with DNA metabolic processes, suggesting that Ftx silencing might impede DNA replication and thereby inhibit CRC cell proliferation. In addition, epithelial-mesenchymal transition (EMT), characterized by loss of E-cadherin, plays a critical role in CRC metastasis [39]. It has been reported that the overexpression of Ftx promotes the migration and invasion of osteosarcoma cells through the ECM mechanism, as evidenced by the loss of E-cadherin and the gain of N-cadherin [40]. Consistently, our results showed that the upregulated proteins due to Ftx silencing were significantly enriched in “cadherin binding” and contained cadherin and cadherin-like domains, suggesting that Ftx might contribute to CRC metastasis via the EMT mechanism.

This study has some limitations that must be addressed. In the present study, the protein expression profile in highly metastatic HCT116 CRC cells was regulated by the lncRNA Ftx, which might be involved in important biological pathways such as ribosomal pathways and extracellular exosome secretion. The findings provide candidate proteins and signaling pathways for further investigation of the underlying mechanism of CRC metastasis. However, a set of biological functional experiments and quantitative assays are needed to clarify the role of the lncRNA Ftx in the regulation of CRC activities. To verify the biological function of the lncRNA Ftx in CRC, we plan on carrying out cell proliferation assays, cell migration assays, and cell invasion assays in the future to determine the tumor cell activity. The expression of candidate genes and signaling factors will be confirmed in in vitro experiments and CRC rat models. Loss- and gain-of-function approaches will be used to establish a direct role of the given candidate gene or signaling factor in CRC metastasis. We hope that these future studies will describe the complete story of the function of the lncRNA Ftx in CRC pathogenesis and activities.

## Conclusions

In conclusion, for the first time, we demonstrated that the lncRNA Ftx regulates the protein expression profile in highly metastatic HCT116 CRC cells. The differentially expressed proteins in response to Ftx overexpression or silencing are mainly distributed in the cytoplasm or nucleus of HCT116 cells and are associated with multiple biological processes and signaling pathways involved in CRC metastasis. Our findings may provide a variety of candidate proteins and signaling pathways for further investigation of the underlying mechanism of CRC metastasis.

## Data Availability

The datasets generated and analyzed during the current study are available from the corresponding author on reasonable request.

## Abbreviations

CRC: Colorectal cancer
lncRNAs: Long noncoding RNAs
TMT: Tandem mass tag
GO: Gene ontology
MCM: Minichromosome maintenance
RP: Ribosomal protein
EMT: Epithelial-mesenchymal transition
